# AEG-1 knockdown in colon cancer cell lines inhibits radiation-enhanced migration and invasion *in vitro* and in a novel *in vivo* zebrafish model

**DOI:** 10.18632/oncotarget.13155

**Published:** 2016-11-07

**Authors:** Sebastian Gnosa, Alessandra Capodanno, Raghavendra Vasudeva Murthy, Lasse Dahl Ejby Jensen, Xiao-Feng Sun

**Affiliations:** ^1^ Department of Oncology and Department of Clinical and Experimental Medicine, Linköping University, Linköping, Sweden; ^2^ Division of Cardiovascular Medicine, Department of Medical and Health Sciences, Linköping University, Linköping, Sweden

**Keywords:** MTDH, colon cancer, zebrafish, transwell migration and invasion, radiation

## Abstract

**Background:**

Radiotherapy is a well-established anti-cancer treatment. Although radiotherapy has been shown to significantly decrease the local relapse in rectal cancer patients, the rate of distant metastasis is still very high. The aim of this study was to evaluate whether AEG-1 is involved in radiation-enhanced migration and invasion *in vitro* and in a novel *in vivo* zebrafish model.

**Results:**

Migration and invasion were decreased in all the AEG-1 knockdown cell lines. Furthermore, we observed that radiation enhanced migration and invasion, while AEG-1 knockdown abolished this effect. The results from the zebrafish embryo model confirmed the results obtained *in vitro*. MMP-9 secretion and expression were decreased in AEG-1 knockdown cells.

**Materials and Methods:**

We evaluated the involvement of AEG-1 in migration and invasion and, radiation-enhanced migration and invasion by Boyden chamber assay in three colon cancer cell lines and respective stable AEG-1 knockdown cell lines. Furthermore, we injected those cells into zebrafish embryos and evaluated the amount of disseminated cells into the tail.

**Conclusion:**

AEG-1 knockdown inhibits migration and invasion, as well as radiation-enhanced invasion both *in vitro* and *in vivo*. We speculate that this is done via the downregulation of the intrinsic or radiation-enhanced MMP-9 expression by AEG-1 in the cancer cells. This study also shows, for the first time, that the zebrafish is a great model to study the early events in radiation-enhanced invasion.

## INTRODUCTION

Colorectal cancer (CRC) is the third most common cancer in men and the second in women worldwide. It is estimated that CRC is responsible for about 700,000 deaths per year, making it the fourth most common cause of cancer death equivalent to about 8% [1]. Surgery is the mainstay of curative therapies for rectal cancer, and both preoperative and postoperative radiotherapy in combination with chemotherapy could significantly decrease the local relapse in several clinical trials [2]. However, the rate of distant metastasis is still very high, and there are currently no treatments available to reduce this risk [3, 4]. Besides the therapeutic effect, radiation may also promote the malignant behavior of cancer cells. In 1991, Von Essen [5] described the occurrence of invaded cells upon radiation treatment. Since then, several studies have shown that radiation enhances migration and invasion both *in vitro* and *in vivo* in different kinds of tumors [6–11].

Studies in hepatocellular carcinoma and glioblastoma cell lines have shown that the activation of the PI3K/Akt pathway and subsequent NF-kB or mTOR activation upon radiation leads to the secretion of matrix metalloproteinases (MMP), mainly MMP-2 and MMP-9, and consequently to enhanced cell invasion [6, 10, 11]. MMPs are important molecules in cancer cell invasion and metastasis which are secreted into the extracellular space and upon activation, degrade extracellular matrix molecules. However, clinical trials with MMP inhibitors have failed, and it remains a challenging task to achieve therapeutically meaningful outcomes by specifically targeting these molecules [12]. Pathways, not implicating MMPs, involved in radiation-enhanced invasion include IGFR-1 and subsequent PI3K/Akt, RhoA and Rock activation as well as K-Ras and c-Raf [8, 10]. Even though several pathways and molecules have been identified, further research is needed to understand and characterize the exact mechanism of radiation-enhanced invasion to be able to develop specific inhibitors.

We previously showed that increased expression of the astrocyte elevated gene-1 (AEG-1) in rectal cancer patients treated with preoperative radiotherapy was independently related to distant relapse and worse disease-free survival. We speculate that the increased distant recurrence rate after radiation in high AEG-1 expressing tumors could be due to the metastasis promoting properties of AEG-1 [13]. AEG-1, also known as Metadherin (MTDH) and LYRIC, was originally identified as a human immunodeficiency virus-1 - inducible gene in human fetal astrocytes [14]. It was shown that AEG-1 mediates metastasis of mouse breast cancer cells to the lungs [15]. In HeLa, human hepatocellular carcinoma, neuroblastoma, and CREF cells, overexpression of AEG-1 increased the matrix invasion and *in vivo* studies using nude mice xenograft models of human hepatocellular cells showed that the overexpression of AEG-1 resulted in highly aggressive and metastatic tumors [16–19]. In 2006, Lee *et al.* [20] identified the first putative activation pathway for AEG-1, in which AEG-1 is activated by the oncogene Ha-ras through the PI3K/Akt pathway leading to the binding of c-Myc to the AEG-1 promoter and transcriptional activation. So far, several signaling pathways downstream of AEG-1 have been discovered, including the NF-κB [18, 21], the PI3K/Akt [22] and the Wnt pathways [16].

The aim of this study was to mechanistically analyze the role of AEG-1 in radiation-enhanced migration and invasion. We therefore evaluated the involvement of AEG-1 in migration and invasion and the impact of AEG-1 on radiation-enhanced invasion *in vitro* in three colon cancer cell lines. Furthermore, we developed a novel zebrafish invasion model to study *in vivo* radiation-enhanced invasion to confirm our *in vitro* results.

## RESULTS

### AEG-1 is involved in migration and invasion of colon cancer cells *in vitro*

**Figure 1:**
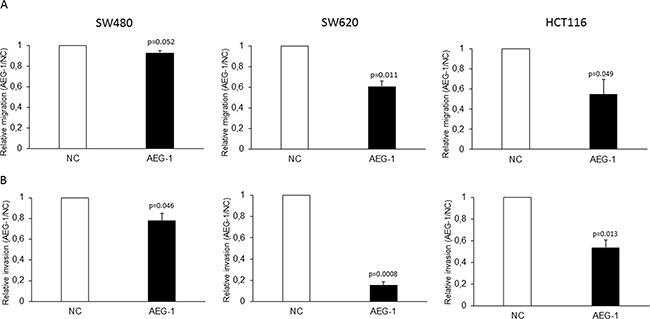
AEG-1 knockdown inhibited migration and invasion in colon cancer cell lines Stable AEG-1 knockdown and negative control cells of the cell lines SW480, SW620 and HCT116 were seeded in FBS free medium in migration chambers or in matrigel invasion chamber respectively, and were placed in 24 well plates with FBS containing medium. After 24 h, migration and invasion were determined by counting the cells migrated or invaded into the membrane. **A.** Stable AEG-1 knockdown cell lines had a lower cell migration compared to the control cell lines SW480 (0.92 fold; p=0.052), SW620 (0.60 fold; p=0.011) and HCT116 (0.55 fold; p=0.049). **B.** Stable AEG-1 knockdown cell lines had a lower cell invasion compared to the control cell lines SW480 (0.78 fold; p=0.046), SW620 (0.15 fold; p=0.0008) and HCT116 (0.53 fold; p=0.013). Data are presented as the ratios between the AEG-1 knockdown and the negative control for each cell line. NC: negative control; AEG-1: AEG-1 knockdown. Error bars represent mean ± SD. All the experiments were performed in duplicates at least three times.

In previous studies, we have shown that AEG-1 was up-regulated in metastases from CRC and that strong expression of AEG-1 was independently correlated to worse distant recurrence- and disease-free survival in patients treated with preoperative radiotherapy, but not in untreated patients [13, 23]. To evaluate whether AEG-1 is involved in radiation-enhanced migration and invasion, we analyzed the role of AEG-1 in migration and invasion in three stable AEG-1 knockdown colon cancer cell lines. These stable cell lines have been established in one of our previous studys [13]. By Boyden chamber assay, we found that AEG-1 knockdown cells had a lower migration compared to the negative control cells in the SW480 (0.92 fold; p=0.052), SW620 (0.60 fold; p=0.011) and HCT116 (0.55 fold; p=0.049) cell lines (Figure 1A). The invasive property of these cell lines were examined by matrigel coated modified Boyden chamber assay. Similar to the migration assay, AEG-1 knockdown cells of all the three cell lines had a significantly lower invasion compared to negative control cells SW480 (0.78 fold; p=0.046), SW620 (0.15 fold; p=0.0008) and HCT116 (0.53 fold; p=0.013) (Figure 1B). The results showed an involvement of AEG-1 in migration and invasion in all the three stable AEG-1 knockdown colon cancer cell lines.

### Radiation enhances migration and invasion in colon cancer cell lines *in vitro*

**Figure 2:**
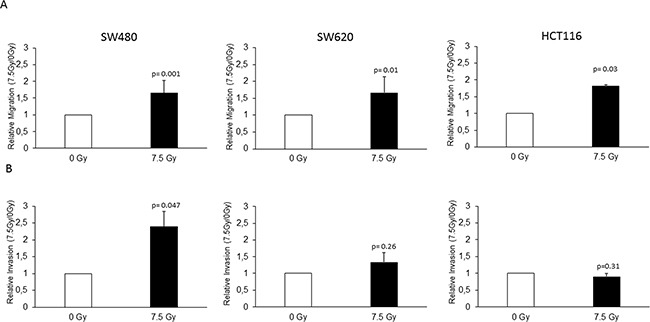
Radiation enhanced migration and invasion of colon cancer cell lines The SW480, SW620 and HCT116 cell lines were seeded in FBS containing medium in migration chamber or in matrigel invasion chamber respectively, placed in 24 well plates with FBS containing medium and either irradiated with 7.5 Gy or unirradiated as a control. After 24 h, migration and invasion were determined by counting the cells migrated or invaded into the membrane. **A.** Radiation-enhanced migration was observed in all the three cell lines, SW480 (1.81 fold; p=0.001), SW620 (1.66 fold; p=0.01), and HCT116 (1.65 fold; p=0.03). **B.** Radiation-enhanced invasion was observed in the SW480 cell line (2.39 fold: p=0.047), a trend in the SW620 (1.33 fold; p=0.26), but not in the HCT116 cells (0.89 fold; p=0.31). Data are presented as the ratios between 7.5 Gy and 0 Gy for each cell line. NC: negative control; AEG-1: AEG-1 knockdown. Error bars represent mean ± SD. All the experiments were performed in duplicates at least three times.

To evaluate the potential of radiation in enhancing metastasis, we analyzed the migration and invasion in the three colon cancer cell lines by Boyden chamber assay. To evaluate the effect of radiation alone, we did not implement a FBS gradient in those respective experiments. A significant increase in migration upon radiation was found in the SW480 (1.81 fold; p=0.001), SW620 (1.66 fold; p=0.01) and HCT116 (1.65 fold; p=0.03) cell lines (Figure 2A). Invasion increased upon radiation only in the SW480 cell line (2.39 fold: p=0.047), while a trend was present in the SW620 cell line (1.33 fold; p=0.26) (Figure 2B). No radiation-enhanced invasion could be seen in the HCT116 cell line (0.89 fold; p=0.31) (Figure 2B). Taken together, we found radiation-enhanced migration in all the three analyzed colon cancer cell lines and radiation-enhanced invasion in the SW480 cell line.

### AEG-1 is involved in radiation-enhanced migration and invasion of colon cancer cell lines *in vitro*

**Figure 3:**
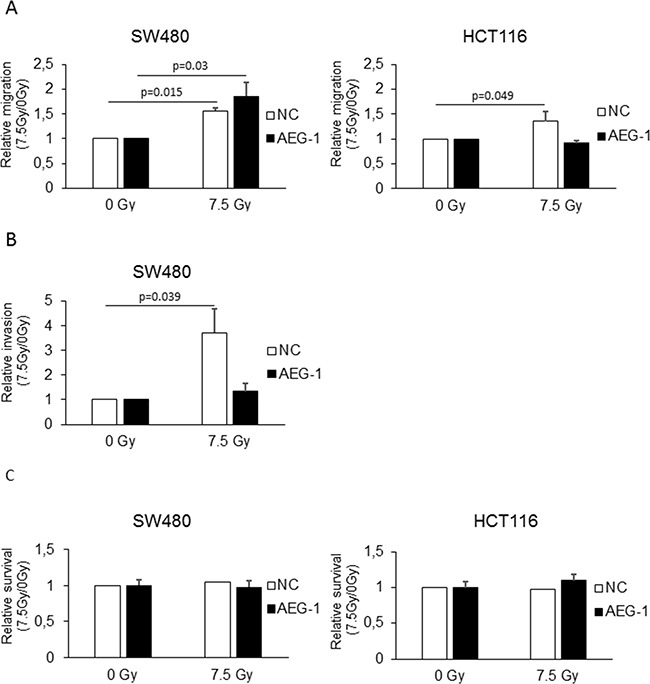
AEG-1 was involved in radiation-enhanced migration and invasion in colon cancer cell lines Stable AEG-1 knockdown and negative control cells of the SW480 and HCT116 cell lines were seeded in FBS containing medium in migration chamber or in matrigel invasion chamber respectively, placed in 24 well plates with FBS containing medium and either irradiated with 7.5 Gy or unirradiated as a control. After 24 h, migration and invasion were determined by counting the cells migrated or invaded into the membrane. **A.** Migration significantly increased upon radiation in both the SW480 negative control (1.56 fold; p=0.015) and SW480 AEG-1 knockdown (1.85 fold; p=0.03) cell lines. In the HCT116 cell line, migration was increased only in the negative control cell line (1.36 fold; p=0.049) whereas in the AEG-1 knockdown cell line the migration rate did not change (0.92 fold; p=0.077) **B.** Invasion significantly increased upon radiation in the SW480 negative control cell line (3.71 fold; p=0.039), but not in the AEG-1 knockdown cell line (1.34 fold; p=0.27). Data are presented as the ratios between 7.5 Gy and 0 Gy for each cell line. NC: negative control; AEG-1: AEG-1 knockdown. Error bars represent mean ± SD and only significant p-values are presented (p<0.05). All the experiments were performed in duplicates at least three times.

To examine whether AEG-1 knockdown could inhibit radiation-enhanced migration and invasion, we evaluated the migration of the SW480 and HCT116 AEG-1 knockdown and negative control cells in response to radiation. The results showed a significant increase in the migration of the negative control cells in both the SW480 (1.56 fold; p=0.015) and HCT116 (1.36 fold; p=0.049) cell lines, similar to the non-transfected cells (Figure 3A). Radiation did not increase the migration in the HCT116 AEG-1 knockdown cells (0.92 fold; p=0.077), while the SW480 AEG-1 knockdown cells showed an increase upon radiation (1.85 fold; p=0.03). Since radiation-enhanced invasion was seen only in the SW480 cell line, we evaluated the impact of AEG-1 knockdown on radiation-enhanced invasion in the SW480 AEG-1 knockdown and negative control cells. In the negative control cells, we observed a significant increase in invasion upon radiation (3.71 fold; p=0.039), similar to the non-transfected cells (Figure 3B). The invasion in the AEG-1 knockdown cells was only slightly increased upon radiation without reaching significance (1.34 fold; p=0.27) (Figure 3B). The results were confirmed in a different AEG-1 knockdown clone with the same AEG-1 shRNA and, in a clone with a different AEG-1 shRNA (data not shown). To evaluate whether the reduced amount of migrating and invading cells upon radiation was due to reduced cell viability, we performed a WST-1 proliferation assay. In both, the SW480 and HCT116 cells, there was no reduction in cell viability 24 h after radiation in the AEG-1 knockdown cells compared to the negative control cells (Figure 3C). These results suggest that AEG-1 knockdown can inhibit the process of radiation-enhanced metastasis.

### Embryonic zebrafish model confirms the involvement of AEG-1 in radiation-enhanced invasion *in vivo*

**Figure 4:**
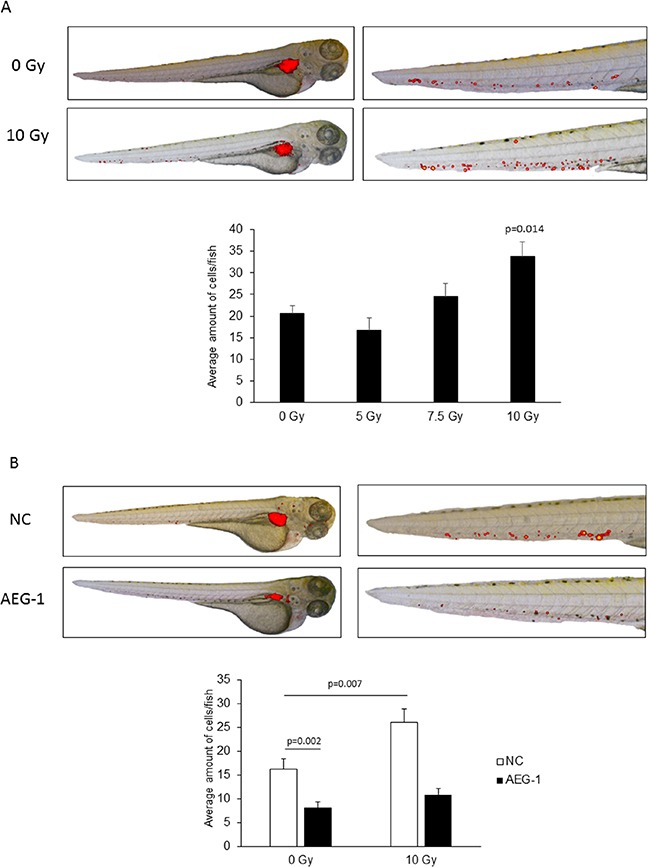
Radiation enhanced invasion via AEG-1 in a zebrafish model DiI labelled, stable AEG-1 knockdown and negative control cells of the SW480 cell line were injected into the perivitelline space of 48 h old zebrafish embryos and irradiated with 0-10 Gy. After incubation for 24 h, the numbers of cells posterior to the anal opening were manually counted. **A.** Dose dependent increase of invaded SW480 cells with a significant invasion after 10 Gy radiation (p=0.014). **B.** AEG-1 knockdown cells had a significant lower cell invasion compared to the unirradiated negative control cells (p=0.002). Radiation increased the invasion significantly upon 10 Gy radiation in the negative control cells (p=0.007) whereas no increase was seen in the AEG-1 knockdown cells. The total numbers of embryos for each group were at least 30. Data are presented as the average number of cells/embryo for each cell line ± SEM. Only significant p-values (p<0.05) are presented. NC: negative control; AEG-1: AEG-1 knockdown.

To further investigate the process of radiation-enhanced invasion, we developed an embryonic zebrafish model for examining the effects of radiation on early stages of tumor cell invasion and dissemination. Firstly, DiI labeled non-transfected SW480 cells were injected into the perivitelline cavity of 48 h old zebrafish embryos, and immediately irradiated with 0-10 Gy. After incubating for 24 h, the amount of disseminated cells into the tail was examined. We found a dose dependent increase of disseminated cells into the tail, with a significant increase in the embryos irradiated with 10 Gy (p=0.014) (Figure 4A). No morphological difference was observed between the unirradiated and irradiated embryos.

Secondly, SW480 AEG-1 knockdown and negative control cells were injected into the zebrafish embryo and irradiated with 0 Gy and 10 Gy. The amount of disseminated cells into the tail of the fish was significantly lower in the AEG-1 knockdown cells compared to the negative control cells 24 h after injection in the unirradiated embryos (p=0.002) (Figure 4A).

As for the non-transfected cells we found in the SW480 negative control cells a significant increase of disseminated cells into the tail in the irradiated embryos compared to the unirradiated control embryos (p=0.007) (Figure 4b). However, there was only a small and non-significant increase upon radiation for the SW480 AEG-1 knockdown cells compared to the unirradiated control (p=0.13). The results from the zebrafish embryo model confirm our results obtained *in vitro*, whereby the invasion can be enhanced by radiation and AEG-1 knockdown can inhibit this process.

### AEG-1 knockdown inhibits radiation-enhanced MMP 9 secretion/expression

**Figure 5:**
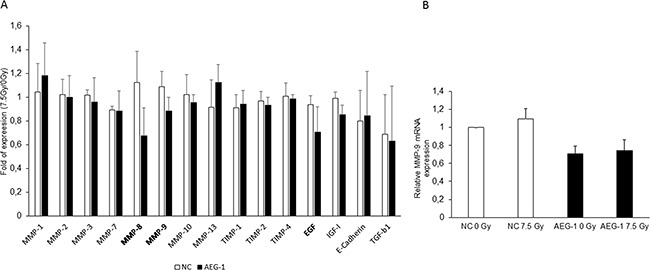
Cytokine array revealed that MMP-8, MMP-9 and EGF were decreased upon radiation in AEG-1 knockdown colon cancer cell lines Conditioned medium of negative control and AEG-1 knockdown cell lines were tested for MMP and cytokine secretion upon radiation by using an antibody array. **A.** MMP-8, MMP-9 and EGF were decreased in the irradiated AEG-1 knockdown cells compared to the unirradiated control, whereas there was a small increase or no change in the negative control cell line. Data presented as the ratios between the 7.5 Gy and 0 Gy for each cell line. **B.** MMP-9 mRNA expression analyzed by qPCR was decreased in SW480 AEG-1 knockdown cells compared to negative control cells. NC: negative control; AEG-1: AEG-1 knockdown. Error bars represent mean ± SD. All the experiments were performed at least three times.

Both MMP-2 and MMP-9 have been shown to be up-regulated after radiation in hepatocellular carcinoma and glioblastoma [6, 24]. Furthermore, it has been shown that AEG-1 knockdown inhibits invasion and decreases the MMP-9 expression in colon cancer cell lines [25]. To evaluate whether MMPs are secreted via AEG-1 after radiation, we performed a cytokine antibody array including MMPs and MMP inhibitors, growth factors and receptors and EMT marker to evaluate possible downstream effectors of AEG-1. The results showed that MMP-8, MMP-9 and EGF were decreased in the irradiated SW480 AEG-1 knockdown cells compared to the unirradiated control while in the SW480 negative control cell line, these factors increased slightly or did not change upon radiation (Figure 5A). To evaluate whether AEG-1 influences the secretion or the expression, we analyzed the MMP-9 mRNA expression after radiation. The results showed a decreased expression of MMP-9 in the SW480 AEG-1 knockdown cells compared to the negative control cells. However, radiation had no effect on the MMP-9 expression in either cell line (Figure 5B).

## DISCUSSION

The present study is the first to evaluate the involvement of AEG-1 in radiation-enhanced migration and invasion. Also, we are the first to use the zebrafish embryo as a model to study radiation-enhanced invasion.

We and others found that the protein and mRNA expression of AEG-1 is higher in metastatic lesions and in primary tumors from patients with liver or lymph node metastasis compared to the primary tumor from patients without metastasis [23, 26–28]. It has been shown that knockdown of AEG-1 inhibits the ability to migrate and invade, whereas AEG-1 overexpression or treatment with TNF-α enhances migration and invasion in different cancer cell lines including colon cancer cell lines [16–19, 28]. In a previous study, we found that tumors expressing high levels of AEG-1 correlate to a higher risk of developing distant metastases in rectal cancer patients treated with preoperative radiotherapy, but not in those without preoperative radiotherapy [13]. We speculate that these findings might be due to the metastasis promoting properties of AEG-1 and the involvement of AEG-1 in radiation-enhanced migration and invasion. In the present study, we confirmed the involvement of AEG-1 in cellular migration and invasion in three stable AEG-1 knockdown colon cancer cell lines.

Both migration and invasion are important for cancer progression and metastasis, and several studies indicated that radiation could enhance the invasiveness of tumor cells [6–10]. However, data about radiation-enhanced migration and invasion in colon cancer are rare and inconclusive. In two studies, it was shown that cellular migration was diminished in the HCT116 cell line in a dose dependent manner [29, 30]. Another study showed that out of three colon cancer cell lines only the HCT116 cell line had an increased matrigel invasion upon radiation with 4 Gy [11]. We evaluated whether the migration and/or invasion were enhanced in any of the three colon cancer cell lines SW480, SW620 or HCT116. Our results showed increased migration upon radiation in all the three colon cancer cell lines. Furthermore, a significant increase in invasion upon radiation was found in the SW480 cell line.

Having shown that AEG-1 was involved in cellular migration and invasion, and that radiation enhanced migration and invasion in colon cancer cell lines, we further investigated the AEG-1 involvement in radiation-enhanced migration and invasion. For the first time, we found that AEG-1 knockdown inhibited radiation-enhanced migration and invasion in colon cancer cell lines. Since radiation-enhanced migration was diminished only in one out of two tested cell lines we speculate that there might be other pathways involved, which are independent of AEG-1.

Many studies that analyze the effect of radiation on invasion are performed *in vitro* since the classical *in vivo* metastasis models performed in mice is high in costs and experimental duration [31–33]. It is furthermore difficult to study the early stage of invasion and metastasis and small metastatic lesions are extremely hard to evaluate in the mouse model [34]. The zebrafish model has been used before to study the metastatic potential and the influence of hypoxia on metastasis on different cell lines and experimental setups [35–38].

In the present study, a zebrafish model was used for the first time to study radiation-enhanced invasion. We used the SW480 cells which showed radiation-enhanced invasion *in vitro* for the zebrafish invasion assay. The results revealed an increased amount of cells invading upon radiation, and decreased cell invasion in the SW480 AEG-1 knockdown cells compared to the negative control cells. Furthermore, there was a reduced radiation-enhanced invasion in the SW480 AEG-1 knockdown cells. The zebrafish model has several advantages compared to the classical mouse model. The amount of offspring's is large, zebrafish embryos are transparent and show no immune reaction at early embryonic stages, and only small numbers of cancer cells are needed for injection [38]. We therefore believe that the zebrafish model is a great supplementation to the already existing models, especially to study early events of radiation-enhanced invasion.

In hepatocellular carcinoma it was shown that radiation enhances invasion via PI3k/Akt, NF-κB and subsequently MMP-9 activation [6]. MMP-9 activation was also found when AEG-1 was up-regulated, and ChIP assay revealed that AEG-1 interacts with the MMP-9 promoter, either via NF-κB or AP-1 [39]. In the present study, we found a decreased expression and secretion of MMP-9 in the SW480 AEG-1 knockdown cells compared to the SW480 negative control cells. Radiation increased the MMP-9 secretion in the negative control cell lines, but not in the AEG-1 knockdown cells. Furthermore, AEG-1 knockdown inhibited the MMP-9 mRNA expression, suggesting that AEG-1 knockdown inhibits invasion and radiation-enhanced invasion possibly via the down-regulation of MMP-9.

In conclusion, our results demonstrate that AEG-1 knockdown can inhibit migration and invasion as well as radiation-enhanced migration and invasion. The novel zebrafish model showed consistent results and represents a great model to study early events in radiation-enhanced invasion.

## MATERIALS AND METHODS

### Colon cancer cell lines and cell culture

The SW480 and SW620 colon cancer cell lines were obtained from the American Type Culture Collection. The cells were maintained at 37°C and 5% CO_2_ in Eagles MEM (Sigma-Aldrich, St. Louis, MO), supplemented with 10% heat inactivated fetal bovine serum albumin (GIBCO, Invitrogen, Paisley, UK) and 2 mM L-Glutamine (Life Technologies, Carlsbad, CA). The HCT116 cell line was obtained from the core cell centre (Johns Hopkins University, Baltimore, MD) and was maintained in McCoy's 5A medium (Sigma-Aldrich) supplemented with 10% heat inactivated fetal bovine serum albumin (GIBCO) at 37°C and 5% CO_2_. Stable AEG-1 knockdown cells (MTDH D) and the corresponding negative control (NC) cells, SW480 NC #6, SW480 MTDH D #10, SW620 NC #2, SW620 MTDH D #2, HCT116 NC #1 and HCT116 MTDH D #3, were established at our laboratory as previously described and used for all the experiments if not differently indicated [13]. The AEG-1 knockdown cell lines and negative control cell lines were cultured under low selection pressure (Pyrumycin 0.1 μg/ml, GIBCO). The AEG-1 protein expression was regularly evaluated by Western blot (Supplementary Figure S1) and qPCR, to confirm AEG-1 knockdown. Cells growing exponentially were harvested when 80% confluence was achieved. All the cells were tested for *Mycoplasma* by using a commercially available PCR kit (PromoKine, Heidelberg, Germany). The morphology and growth rate of all the cell lines were controlled during the whole experimental period.

### Zebrafish breeding

All the zebrafish experiments were approved by the institutional ethics committee at Linköping University. Transgenic Tg(fli1:EGFP)^y1^ zebrafish (ZIRC, Eugene, OR), with EGFP labeled endothelial cells [35] were maintained according to standard protocols at the zebrafish facility at Linköping University. Zebrafish embryos were produced by natural mating, and eggs were collected in the morning immediately after spawning. Subsequently, the eggs were cleaned, sorted for successful fertilization and incubated in E3 medium supplemented with 1-phenyl-2-thiourea at 28.5°C in humidified ambient air until injection.

### Ionizing radiation

To determine the effect of ionizing radiation, the cells and zebrafish embryos were irradiated with a 6 MV photon spectra using a linear accelerator (Clinac 4/100, Varian, Palo Alto, CA). The cells were positioned below 3 cm PMMA, 105 cm from the photon source (the distance from the photon source to the PMMA-surface was 100 cm). The dose rate at the position of the cells was 4.8 Gy/min and the field size at SSD was 30 x 30 cm.

### Boyden chamber migration and modified Boyden chamber matrigel invasion assay

Boyden chamber migration assays (8 μm pore size, Corning, NY) and modified Boyden chamber matrigel invasion assays (8 μm pore size, Corning) were performed according to the manufacturer's instructions. Briefly 1 x 10^5^ (SW480 and HCT116) or 2 x 10^5^ (SW620) cells were seeded into the migration or matrigel invasion chamber, which were placed in a 24 well plate containing complete medium and incubated at 37°C and 5% CO_2_ for 24 h. To evaluate the effect of AEG-1 knockdown, the cells were seeded in FBS free medium. To evaluate the effect of radiation, the cells were seeded in complete medium, followed by radiation with 0 Gy or 7.5 Gy within 1 h after seeding and incubated at 37°C and 5% CO_2_ for 24 h. After incubation the cells were washed with PBS, fixed in 4% para-formaldehyde and stained with 0.2% crystal violet in 2% ethanol. Non-invaded cells in the upper chamber were removed using a cotton swab. The membranes were moved from the chamber and fixed at a cover slide. Images of migrated or invaded cells where taken under a light microscope (Zeiss Lab.A1, Jena, Germany). Invasion and migration was determined by counting the cells migrated and invaded trough the filter with x-100 magnification.

### Zebrafish experiments

The cell lines were labelled with 1,1’-dioctadecyl-3,3,3’3’-tetramethylindocarbocyanine (DiI) at a concentration of 5 ng/ml in PBS for 1.5 h, re-plated in complete medium and incubated at 37°C and 5% CO_2_ for 24 h. After labelling, the cells were collected and injected into the perivitelline space of 48 h old embryos, as previously described [36]. After injection, the embryos with labeled cells in the circulation were excluded. The amount of injected cells was identical for all treatment groups and showed only a slight variation between the embryos. The embryos were irradiated with 0-10 Gy and incubated at 28.5°C in humidified ambient air. After 24 h, the embryos were anaesthetized with 0.004% triciane and the cells were visualized under a fluorescence microscope (Nikon D-eclipse C1, Tokyo, Japan). The cells presented posterior to the anal opening were counted manually in at least 30 embryos per treatment group.

### Cell proliferation assay

The proliferation of the different cell lines upon radiation was measured by the WST-1 assay (Roche, Basel, Switzerland) according to the manufacturer's instructions. Briefly, 10,000 cells (SW480) or 3,000 cells (HCT116) were seeded in 100 μl complete medium in 96 well plates 24 h before radiation and incubated at 37°C and 5% CO_2_. The proliferation was determined 24 h after radiation with 0 Gy or 7.5 Gy.

### Cytokine array

The cytokine antibody array (custom c-series cytokine array, RayBiotech, Norcross, GA) contained 15 antibodies, including 8 MMPs, 3 MMP inhibitors, 3 growth factors, and E-cadherin. The array was performed according to the manufacturer's instructions. Briefly, 1 x 10^5^ cells in 2 ml complete medium were seeded in 6 well plates 24 h before radiation and incubated at 37°C and 5% CO_2_. One hour before radiation the medium was replaced by 1.5 ml serum free medium and, 24 h after radiation the medium was collected and stored at -80° C until further usage.

Each membrane was incubated with 1 ml conditioned medium over night at 4°C and subsequently washed and incubated with a biotinylated antibody cocktail for 2 h at room temperature. The membranes were washed and incubated for 2 h with HRP-Streptavidin and the proteins were detected using chemiluminescence and visualized by CCD camera (LAS-100, Fujifilm, Tokyo, Japan). ImageJ software was used to quantify the protein expression. The ratio between unirradiated and irradiated was compared between AEG-1 knockdown and negative control cells and we considered a difference of 20% to be a change in expression level.

### qPCR

The mRNA expression levels of MMP-9 were determined in the SW480 AEG-1 knockdown and negative control cells after radiation using the TaqMan™^®^ Gene Expression Fast Master Mix in the Applied Biosystems 7900HT Fast Real-Time PCR System according to the manufacturer's instructions. All samples were performed in triplicates and normalized to *GAPDH*. Primers and hydrolysis probes were TaqMan™^®^ Gene Expression assays on demand for *MMP-9* (Hs00234579_m1) and *GAPDH* (4352934E) (Applied Biosystems, Foster City, CA). The PCR amplification program was the following: denaturation (95°C 20 sec), amplification and quantification (95°C 1 sec and 60°C 20 sec for 40 cycles). In addition, ddH_2_O and a minus RT product as negative controls were analyzed for every plate. Relative quantification and fold expression changes were determined by the 2^-ΔΔCt^ method.

### Statistical analysis

All the experiments were repeated at least three times, if not indicated differently and presented as mean ± standard deviation (SD), or standard error of the mean (SEM). Student's t-test was applied for statistical significance. All the tests were two sided, and p-values less than 0.05 were considered as significant.

## SUPPLEMENTARY MATERIALS FIGURE


