# Supplementary material for: AEG-1 knockdown in colon cancer cell lines inhibits radiation-enhanced migration and invasion in vitro and in a novel in vivo zebrafish model
Source: Oncotarget. 2016 Nov 7;7(49):81634–44. doi: 10.18632/oncotarget.13155 (PMC5348418; doi:10.18632/oncotarget.13155)
Supplement: Supplementary file 1 [file oncotarget-07-81634-s001.pdf]

AEG-1 knockdown in colon cancer cell lines inhibits radiation-enhanced migration and invasion *in vitro* and in a novel *in vivo* zebrafish model

SUPPLEMENTARY FIGURE

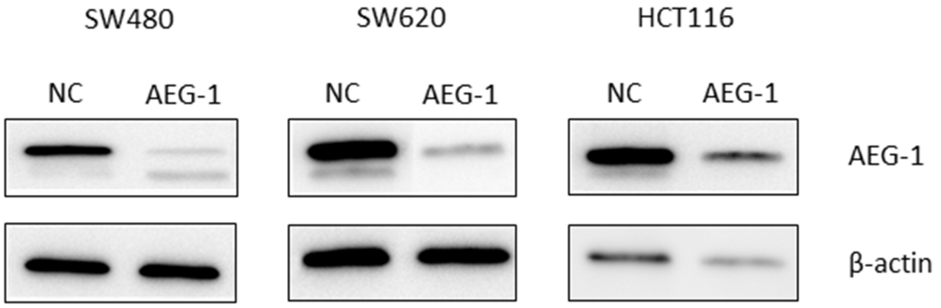

Supplementary Figure S1: AEG-1 expression in stable knockdown and negative control cell lines. The AEG-1 protein expression examined by Western blot from stable AEG-1 knockdown and negative control cell lines: SW480, SW620 and HCT116. β-actin was used as a loading control. NC: negative control; AEG-1: stable AEG-1 knockdown.
